# Emerging Role of PACAP as a New Potential Therapeutic Target in Major Diabetes Complications

**DOI:** 10.1155/2015/160928

**Published:** 2015-05-14

**Authors:** Rubina Marzagalli, Soraya Scuderi, Filippo Drago, James A. Waschek, Alessandro Castorina

**Affiliations:** ^1^Section of Human Anatomy and Histology, Department of Biomedical and Biotechnological Sciences, University of Catania, 95123 Catania, Italy; ^2^Section of Pharmacology, Department of Biomedical and Biotechnological Sciences, University of Catania, 95123 Catania, Italy; ^3^Semel Institute, Department of Psychiatry, David Geffen School of Medicine, University of California Los Angeles, Los Angeles, CA 90095, USA

## Abstract

Enduring diabetes increases the probability of developing secondary damage to numerous systems, and these complications represent a cause of morbidity and mortality. Establishing the causes of diabetes remains the key step to eradicate the disease, but prevention as well as finding therapies to ameliorate some of the major diabetic complications is an equally important step to increase life expectancy and quality for the millions of individuals already affected by the disease or who are likely to develop it before cures become routinely available. In this review, we will firstly summarize some of the major complications of diabetes, including endothelial and pancreatic islets dysfunction, retinopathy, and nephropathy, and then discuss the emerging roles exerted by the neuropeptide pituitary adenylate cyclase activating polypeptide (PACAP) to counteract these ranges of pathologies that are precipitated by the prolonged hyperglycemic state. Finally, we will describe the main signalling routes activated by the peptide and propose possible future directions to focus on developing more effective peptide-based therapies to treat the major complications associated with longstanding diabetes.

## 1. Introduction

The prevalence of diabetes is increasing worldwide. According to the World Health Organization (WHO), the total number of people affected by diabetes is expected to rise to an estimated 300 million cases by the year 2025 [1]. The onset of diabetes immediately increases the possibility for the patient to develop a broad spectrum of irreversible complications [2]. Both type 1 [3] and type 2 [4] diabetes have devastating consequences especially on small and large blood vessels. A considerable effort has been put into finding treatments for this condition and its complications. Among them, targeting endogenous peptidergic systems or their downstream signalling mechanisms is emerging as a valuable therapeutic option.

In this review we will outline some of the most recent advances from our research group and others in relationship to the role of a promising neuropeptide candidate endowed with potential beneficial effects to treat or ameliorate certain aspects of diabetes, namely, the pituitary adenylate cyclase-activating polypeptide (PACAP). We will emphasize how the neuropeptide interferes and in some cases prevents the development of specific pathological consequences of the disease, such as micro- and macroangiopathy, retinal dysfunction, and defective pancreatic *β*-cell insulin secretion, and discuss the major intracellular events implicated and how these are hampered by peptide treatment. The final purpose will be to shed more light into PACAP function for future exploitation in therapies aimed at arresting or preventing the development of complications associated with disease progression.

## 2. Diabetes and Its Complications

The term diabetes mellitus indicates a group of metabolic disorders characterized by high blood sugar and impaired insulin signaling. The disease is usually classified into type 1 diabetes, type 2 diabetes, and other specific types including gestational diabetes. Type 1 diabetes, also known as insulin-dependent diabetes, represents about 5–10% of all cases of diabetes. It is characterized by *β*-pancreatic cells destruction that leads to absolute insulin deficiency. This failure to produce insulin can be autoimmune-mediated or idiopathic. Type 2 diabetes constitutes 90–95% of all diabetes cases and results from insulin resistance, inadequate compensatory insulin secretory response, or both. It is characterized by reduced responsiveness of peripheral cells in the body to insulin and, consequently, reduced ability to transfer glucose out of the circulation [5]. Complications of diabetes can be largely divided into microvascular and macrovascular complications. The microvascular complications include diabetic retinopathy, diabetic neuropathy, and diabetic nephropathy. The macrovascular complications include cerebrovascular disease, coronary heart disease, and peripheral vascular disease (summarized in Table 1).

Elevated glucose levels result in an increased production of reactive oxygen species (ROS) and inflammatory mediators strictly involved in vasculature dysfunction. The altered system not only causes hypertension, ischemia, and/or altered vascular permeability but also contributes to other complications of diabetes [6, 7].

In the last decades, a big effort was put into finding new therapeutic approaches to target either the full range of diabetic complications or the damaged tissues/organs. Although several classes of antidiabetic drugs are currently available, achieving and maintaining long-term glycemic control are often challenging and not sufficient alone, so a significant need for novel antidiabetic drugs remains. It has been discovered that neuropeptides and their analogues, activating different signalling pathways through their receptors, are emerging as new therapeutic options. Among these, one of the most promising human peptides is the pituitary adenylate cyclase-activating polypeptide (PACAP). This review outlines the knowledge accumulated over the past years on the role of this neuropeptide in the major complications of diabetes, particularly emphasizing its potential for new therapeutic approaches.

## 3. PACAP, Receptors, and Functions

**Figure 1:**
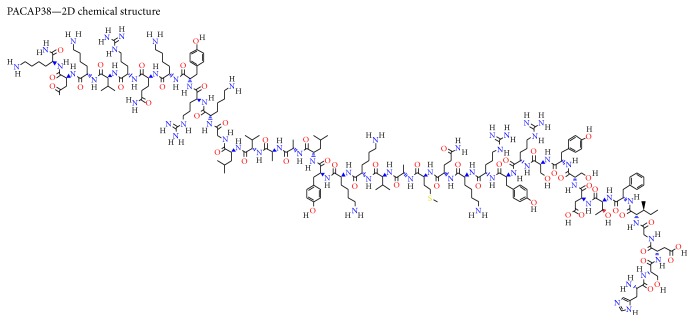
2D chemical structure of full-length PACAP38 (CID 44566111). The chemical structure was retrieved using the publicly available PubChem database source, accessible at https://pubchem.ncbi.nlm.nih.gov/compound/44566111#.

PACAP (molecular formula C_203_H_331_N_63_O_53_S; for details on PACAP38 chemical structure please refer to Figure 1) was originally isolated from an ovine hypothalamus extract [8]. Two PACAP isoforms have been identified, a 38-amino-acid form (PACAP38) and a C-terminally truncated 27-amino-acid form (PACAP27). It belongs to the secretine, glucagon, and peptide histidine-isoleucine (PHI) superfamily and binds to an overlapping group of receptors. PACAP receptors are G protein-coupled receptors and can be classified into two main groups, PAC1 and VPAC (including VPAC1 and VPAC2 subtypes), both of which activate adenylate cyclase with consequent stimulation of cAMP production and IP accumulation [9, 10]. In distinct cell types, PACAP is able to trigger different pathways, depending on which receptor splice variants are expressed, peptide concentration being used, and other biological factors. Among PACAP functions, the one that mostly emerges is its strong cytoprotective role, promoting survival in many types of neuronal and nonneuronal cells and tissues, including lymphocytes, chondrocytes, endothelial cells, Schwann cells, liver, lung, and ovary [11–17].* In vitro* and* in vivo* studies have shown that the peptide acts through the PAC1 receptors to stimulate various downstream executors of the protein kinase A and C (PKA and PKC) pathways [18–21]. It also activates ion channels, *β*-arrestin signalling, and mitogen-activated protein (MAP) kinase in some tissues [22, 23]. PACAP and its receptors have been detected in multiple organs, with the highest levels in endocrine glands and in the central nervous system. Lower expression has been identified in gastrointestinal, respiratory, cardiovascular, and urogenital systems [24, 25]. Ultrastructural studies have also revealed that PACAP immunoreactivity is mainly localized near the plasma membrane, in the rough endoplasmic reticulum, in the Golgi's apparatus, and in the cytoplasmic matrix [26–28]. Such a broad distribution of PACAP in several tissues/organs, together with the variety of signalling pathways shown to be activated, poses the peptide in a position suitable to be considered a key drug candidate with the potential to ameliorate a wide spectrum of disorders, including diabetes and its major complications.

## 4. PACAP and PAC1 Receptors Distribution in the Retina

PACAP and its high-affinity receptor PAC1 are broadly distributed in a number of organs/systems. Of interest, different studies using rodents have shown that both PACAP and PAC1 immunoreactivity (IR) sites are also detectable in the retina and the nervous tunica of the eye, both known to be highly sensitive to long-lasting glucose insult. Indeed, immunohistochemistry studies have shown that PACAP-like IR is evident in a population of sensory neurons in the rat uvea [29, 30] and in trigeminal ganglion cells [31]. PACAP-IR is also localized at high densities in nonretinal parts of the eye, such as the iris sphincter and the ciliary body [31, 32]. Topographically, specific cell populations were found to be positive for PACAP in retinal tissue samples. In particular, PACAP-positive nerve fibers have been detected in the nerve fiber layer (NFL), the ganglion cell layer (GCL), and the inner plexiform layer (IPL). PACAP-IR was found to be positive in neuronal cell bodies of amacrine and horizontal cells localized in the inner nuclear layer (INL). However, no PACAP-IR has ever been established in rods and cones (photoreceptors) in the outer nuclear layer (ONL) or in neighboring retinal pigmented epithelium [27, 33, 34].

In the IPL, PACAP^+^ amacrine cell processes make synaptic contacts with retinal ganglion cell (RGC) terminals, as well as amacrine and bipolar cell processes. PACAP^+^ amacrine cells also establish synaptic contacts with each other. PACAP^+^ axon terminals establish synapses with ganglion cells, bipolar cells, amacrine cells, and horizontal cells in the GCL, NFL, and IPL.

**Figure 2:**
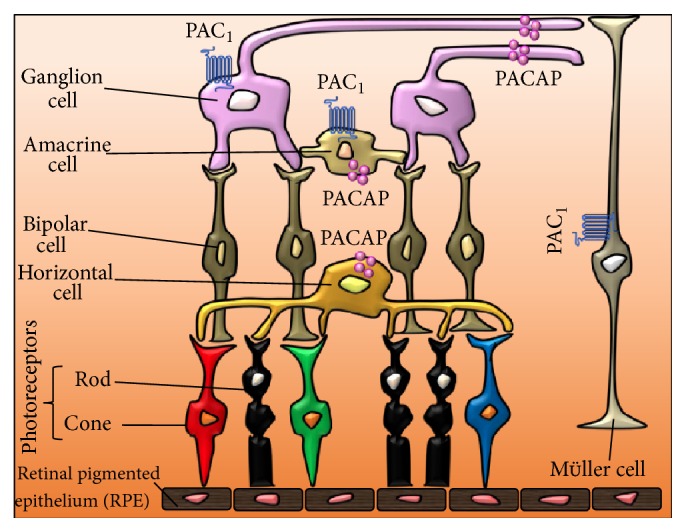
Representative schematic illustration showing PACAP and PAC1 distribution in rodent retina. Immunohistochemical studies have shown that PACAP immunoreactivity (IR) is detectable in nerve fibers (neurites) originating from retinal ganglionic cells as well as in neuronal cell bodies of amacrine and horizontal cells in the inner nuclear layer. No evidence of PACAP-IR has been provided in photoreceptors in the outer nuclear layer (ONL) or in the retinal pigmented epithelium (RPE) [22, 28, 29].

Studies concerning PAC1 receptor distribution have indicated that binding sites for PACAP and VIP, positively coupled to adenylate cyclase, are expressed in the retina of different mammalian species [18]. PAC1-IR in the retina seems to be mainly detectable in the cell bodies and processes of ganglionic and amacrine cells but not in photoreceptors. At the subcellular level, PAC1 receptors are localized at the plasma membrane, in the rough endoplasmic reticulum, and in the cytoplasmic matrix of RGCs and amacrine cells in the INL. Of note, it has been shown that PAC1 receptor localization not always matches with peptide expression. For instance, in the rat brain, PAC1-IR has been found at high levels in the olfactory bulb, hippocampus, and cerebellar cortex, where only few or no PACAP-containing neurons are identified. However, this may be due to the difficulties associated with detecting antigens in retinal tissue or in nervous tissue in general. However, although it has not been detected in Müller cells* in vivo*, PAC1^+^ has been shown in rat primary cultures of Müller cells [35]. For a comprehensive summary depicting PACAP and PAC1 receptor localization to specific cell populations in rodent retina, please refer to Figure 2.

## 5. PACAP and Diabetic Endothelial Dysfunction

Diabetes is characterized by a high risk of developing chronic complications, with micro- and macrovascular impairments in multiple organs. Today it is well established that increased oxidative stress impairs endothelial function and it is thought to mediate vascular disease.

Vascular function depends upon the balance of oxidant and antioxidant mechanisms. During diabetes, an unbalance between anti-inflammatory and antithrombotic homeostasis in favor of proinflammatory and thrombogenic elements is evident. Heightened oxidative stress and altered expression of a variety of genes related to atherogenesis and angiogenesis have also been described in the vasculature exposed to elevated levels of glucose [6, 7, 36–39].

**Figure 3:**
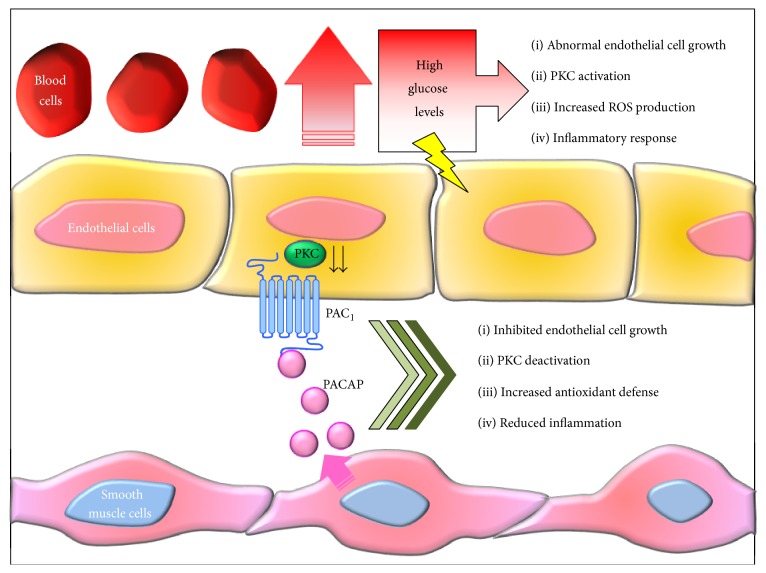
Proposed model depicting the ameliorative role of PACAP in hyperglycemia-induced endothelial dysfunction: potential involvement of smooth muscle cells through a paracrine mechanism. One of the most devastating complications of persistent hyperglycemia is micro- and macroangiopathy that may determine abnormal vasculogenesis. However, the exact pathogenetic mechanisms are still not well understood. In the proposed scenario, longstanding high glucose levels in the blood stream cause both mechanical (shear stress) or oxidative injury (↑ ROS production) to endothelial cells, whose in turn react by triggering local inflammatory responses, sustained PKC activation, and aberrant angiogenesis (↑ endothelial cell proliferation). PACAP seems to play a homeostatic role in this process. Once released by the neighboring smooth muscle cells (SMCs) it binds to PAC1 receptors expressed by both endothelial cells and SMCs, thereby activating a cAMP-driven signalling cascade that inhibits glucose-induced proliferation of both cell populations and ROS production and dampens PKC function as well as local release of proinflammatory cytokines by infiltrating macrophages.

In addition, trophic factor alterations associated with early and late stages of diabetes seem to aggravate hyperglycemia-induced proliferative response, resulting in increased basement membrane thickening and aberrantly regulated vasculogenesis. On the other hand, molecules like PACAP, though being commonly recognized as trophic factors, have demonstrated to also possess opposite biological roles in the vasculature system, most likely depending on the surrounding microenvironment or other coexisting factors, and have been therefore defined as “nonclassic endogenous regulators of angiogenesis” [39]. To support this concept, studies from independent laboratories, including ours, have succeeded to demonstrate that PACAP exerts a rate-limiting effect on cell proliferation under specific pathological conditions replicated* in vitro*, such as longstanding high glucose exposure in murine microvascular endothelial cells [40] or in aortic smooth muscle cells [41], acting oppositely to the canonical trophic roles. However, the seeming duality of PACAP acting either as a “classic” or “nonclassic” trophic factor molecule should be considered with care. The majority of growth-promoting functions associated with PACAP are complex and context specific, suggesting that the way PACAP responds to “stimuli” depends both on the cell/tissue types, local peptide concentration, bioavailability, and alternative splicing mechanisms but depends also on the nature of the insult itself. In our laboratory, we showed that PACAP was apparently devoid of biological activity on endothelial cells up to 7 days of exposure, whereas the peptide elicited an antiproliferative response after chronic exposure (up to 15 days) to high glucose (25 mM) in the H5V murine microvascular endothelial cell line used [42]. In that same study, we also observed that PACAP and its structurally related homolog vasoactive intestinal peptide (VIP) were unable to induce proliferative activity on their own or to show intrinsic effects on cell viability in these cell lines, although they prevented glucose-induced cell death, possibly by dampening ROS-induced oxidative stress, as indicated by other researchers [43]. It is thus plausible that PACAP may play a dual growth-inhibitory and protective role to arrest the aberrantly activated neovascularization and prevent ROS-mediated apoptosis under hyperglycemic conditions (a schematic representation is depicted in Figure 3).

The importance of PACAP in relationship to circulatory dysfunction caused by long-standing diabetes does not seem to be solely limited to restoring microvasculature, as the combined vasodilatory effects together with the growth-inhbitory functions of the peptide can be extended to the circulatory system in general. In particular, the PACAP homolog VIP has captured scientific interest because of its growth inhibitory properties on smooth muscle cells [44], inferring on its potential effects on diabetes-induced hypertension. It was later discovered that, similarly to VIP, a comparable effect can also be achieved using PACAP, since the main receptor involved is the PACAP/VIP equal affinity receptor VPAC2 [41]. Consistent with the hypotensive effect mediated by VPAC2 receptors, at least a study has demonstrated that PACAP, synthesized in the adrenal medulla, is able to activate VPAC1/VPAC2 receptors in the zona glomerulosa of the adrenal gland, ultimately promoting the systemic release of aldosterone, an endogenous hypotensive hormone [45]. However, other important functions that may be transferred to the circulatory system have been observed, in particular concerning VPAC2 receptor function. Of note, evidence has been provided regarding the potential lipolytic effects exerted by PACAP/VIP-VPAC2 axis in cultured adipocytes [46]. Such activity may have positive repercussions for the search of new strategies to counteract atherogenesis, especially in chronic diabetic patients.

Based on the* in vitro* and* in vivo* evidences discussed here, it appears that PACAP, by recruiting different receptors in a cell type-dependent fashion, activates a series of physiological/compensatory responses finalized to reestablish vascular homeostasis. However, further investigations might still be needed to better define the specific actions mediated by each binding receptor, as this will set the stage to develop new highly selective PACAP analogues or receptor agonists encompassing therapeutical activity for the treatment of vascular complications associated with diabetes.

## 6. Trophic Effects of PACAP on Pancreatic Islets 

A hallmark of both types I and II diabetes is the progressive *β*-cell insufficiency in the pancreas, which results in defects in insulin secretion and hyperglycemia [47, 48]. Pancreatic islet cell apoptosis, induced by various factors such as oxidative stress, inflammation, and toxicity of chronic high blood sugar, results inevitably in a decline of *β*-cell mass and function in diabetic patients and causes diabetic complications, including diabetic nephropathy, neuropathy, and retinopathy [49, 50]. About two decades ago, PACAP was identified as a novel islet substance that is synthesized and released by pancreatic *β*-cells. PACAP protein was colocalized to secretory vesicles of insulin and glucagon-containing cells in human and rodent pancreases [51].* In situ* hybridization studies have also shown that both PAC1 and VPAC2 receptors are expressed in the *β* cells, being the predominant PACAP binding receptors in these cells, while VPAC1 receptors were detected only in vessels surrounding the islets [22, 52]. Accumulating* in vivo* and* in vitro* studies have shown that PACAP, in an autocrine and/or paracrine manner, produces trophic effects on *β*-cells. It stimulates the main endocrine pancreatic function (insulin production and release), preserves *β*-cell responses to glucose, and regulates both proliferation and cell viability [53–57]. Studies attempting to establish the exact role of each of the receptors in mediating beneficial activities on *β*-cell population have taken advantage on genetically engineered mice with either a targeted deletion of the PAC1 receptor gene (PAC1−/−) or of the VPAC2 receptor gene (VPAC2−/−). Either knockout animal model displays reduced insulin response to both gastric and, to a lesser extent, intravenous glucose administration. These mice also display impaired glucose elimination. PAC1 deletion is also associated with a robust reduction of glucose-stimulated insulin secretion, suggesting that PACAP through PAC1 receptors might also play a role in modulating the insulin secretory response to glucose itself. Regarding VPAC2−/− models, although these mice develop normally, they exhibit increased metabolic rate and increased insulin sensitivity, accompanied by reduced body weight due to reduced fat mass. As for PAC1 receptors, these observations suggest that VPAC2 receptors might as well be necessary for physiological insulin secretion [58]. However, despite the common insulinotrophic responses mediated by the two receptors, studies focusing on the diverse potential actions exerted by PACAP are still warranted, since a variety of different intracellular signalling molecules (e.g., cAMP, PKA, ion channels, and MAP kinases) may be differently involved and act in concert in a context-dependent manner to produce variable effects on pancreatic islets during diabetes progression.

## 7. PACAP Protection in Diabetic Retinopathy

**Figure 4:**
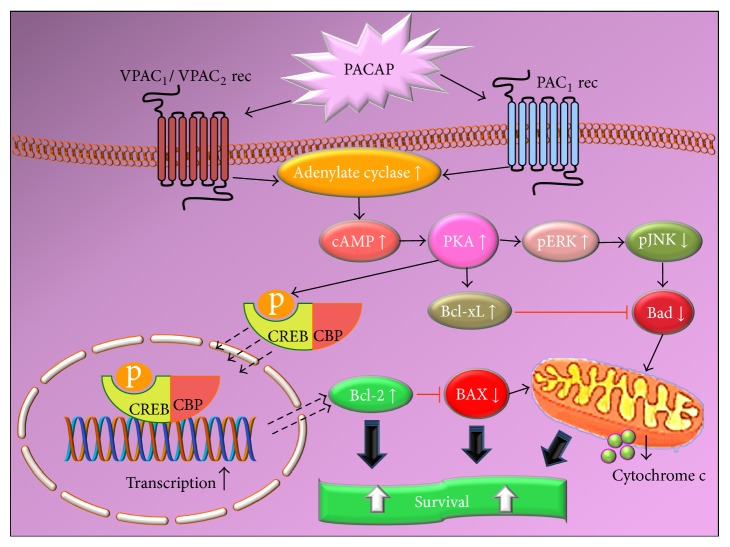
Schematic diagram showing the main prosurvival/antiapoptotic intracellular pathways activated by PACAP. As apparent from the diagram, the intrinsic apoptotic cascade represents one of the main targets of PACAP to protect cells from death. cAMP, cyclic 3′,5′ adenosine monophosphate; PKA, protein kinase A; PAC_1_, VPAC_1_, VPAC_2_, PACAP-binding receptors; ERK, extracellular regulated protein kinase, Bcl-xL, Bcl-2, antiapoptotic factors; JNK, Janus kinase, Bad, BAX, cytochrome c release, proapoptotic factors; CREB, cAMP responsive element binding protein; CBP, CREB binding protein.

Diabetic retinopathy (DR) is one of the most significant and disabling chronic complications of diabetes mellitus [59]. DR can be generally divided into two clinical stages: nonproliferative and proliferative diabetic retinopathy (PDR). During nonproliferative DR, abnormal permeability and/or nonperfusion of capillaries lead to the formation of microaneurysms and leakage of fluid and solutes into the surrounding retinal tissue which accumulates around the macula, causing diabetic macular oedema (DMO). As DR severity increases, vascular abnormalities (occlusion of retinal capillaries; retinal ischemia) and aberrant neovascularization, a process by which new blood vessels proliferate on the surface of the retina, defining PDR, occur [60]. The tight control of blood glucose levels and blood pressure are essential in preventing or arresting their development. However, these therapeutic objectives are difficult to achieve, even with strict glycemic control, and as a consequence PDR and DMO still appear in a high percentage during the evolution of the disease in patients with both type I and type 2 diabetes [61]. DR has been considered, for many decades, as a microangiopathy disease of the retina with key clinical features, vascular leakage, and preretinal neovascularization, resulting from breakdown of the blood retinal barrier (BRB) [62]. However, there is a mounting evidence to suggest that the pathogenesis of DR may also comprise neuroinflammatory and neuropathic processes, which contribute to visual impairment [63]. Loss of neuroretinal adaptation to the diabetic metabolic environment and neural apoptosis may occur in DR prior to any clinically detectable microvasculopathy, in both human and animal models [64]. In this scenario, hyperglycemia is considered the main culprit for both neural and vascular compromising. The numerous metabolic pathways triggered by hyperglycemia such as the polyol pathway, the hexosamine pathway, the DAG-PKC pathway, advanced glycation end-products beside extracellular glutamate accumulation, oxidative stress, and reduction of neuroprotective factors synthesized by the retina lead to neuronal apoptosis and glial dysfunction, hallmarks of retinal neurodegeneration and BRB breakdown, vasoregression, and altered microvascular system [65]. The presence of neuropeptides in the human retina has mostly been studied by immunohistochemical and chromatographic assays. Neuropeptides are produced from both neural and nonneural cells, and some peptides can also be produced by other extraretinal cell sources. It has now become clear that the neuropeptides described in the retina can be divided in two categories: peptides that promote the development of DR symptoms and others that are able to prevent, delay, or eliminate them. The retinal balance between the neurotoxic and neuroprotective events is crucial to determine neuronal cell fate in the diabetic retina [66]. It is well documented that PACAP and its receptors are expressed in the retina [18, 27, 28] and increasing evidence suggests the retinal protective role of the peptide in different retinal pathologies. A recent study by our research group has shown that, in retina of diabetic rats, an initial upregulation of both PACAP and related receptors preceding morphological evidence of cell death is evident [67]. In that same study, we also showed that PACAP intravitreal treatment downregulated the expression of proapoptotic genes along with other evidences of its protective effects, suggesting an active role for this peptide in counteracting some of the pathogenetic events of DR by different mechanisms, including the apoptotic machinery [68]. Additional studies have confirmed that intraocular injection of PACAP exerts protective effects by increasing anti- and decreasing proapoptotic factors. Indeed, it has been shown that PACAP injections markedly attenuated diabetic retinal injury by increasing the levels of the antiapoptotic p-Akt, pERK1, p-ERK2, PKC, and Bcl-2 signalling, while decreasing the levels of the proapoptotic p-p38MAPK pathway (Figure 4). PACAP also protects ganglion cells and dopaminergic amacrine cells degeneration in experimental diabetes [69, 70]. PACAP emerges as a strong antiapoptotic factor that exerts its effect by acting at different levels of the apoptotic cascade [70, 71]. Further, the broad distribution of the peptide, along with that of its high affinity receptor PAC1, raises the question on whether PACAP-PAC1 axis might also play protective functions in the retina. Consistent with previous evidences from our research group [67], Szabadfi and coworkers [68, 69] have shown that retinal PAC1 receptors are the main receptors to mediate the ameliorative effects on the structural changes caused by streptozotocin in a model of early diabetic retinopathy. It appears though that PACAP acting through PAC1 may indeed trigger protective mechanisms in the retina during diabetic insults. Consistent with such protective function, its potential therapeutic role has also been demonstrated in a model* in vitro* of DMO, hence showing that PACAP is able to also prevent the disruption of the BRB, mainly by modulating the expression of important tight junctions such as* zona occludens-1* (ZO-1) and claudin-1, both essential for the proper functionality of the retinal barrier during diabetes [72].

## 8. PACAP in Diabetic Nephropathy

Alterations in renal function and structure are found even at the onset of diabetes mellitus. Diabetic nephropathy is characterized by initial proteinuria followed by a decline in glomerular filtration rate and ultimate progression to uraemia. Diabetic nephropathy is the leading cause of end-stage renal failure and about 30–40% of patients need renal transplantation [73]. The main clinical features that frequently precede diabetic nephropathy are hypertension and poor glycemic control. Key factors that are involved in diabetic kidney damage are oxidative stress, overproduction of advanced glycation end products (AGE), apoptosis, and inflammation due to the local release of proinflammatory cytokines [74]. Despite the well-established activities of the peptide in many other diabetes complications, the action of PACAP in the kidney of diabetic patients has captured scientific interest only in the past two years. Since the pleiotropic peptide is known to exert anti-inflammatory, antiapoptotic, and antioxidant effects, it appeared reasonable to think that it could be a suitable candidate to also prevent the development or delay the progression of DN. A recent publication by Banki and coworkers identified new molecular mechanisms underlying PACAP nephroprotective properties, including the ability to reduce fibrotic markers, like collagen IV and TGF-*β*1 in the kidney, or hampering the proapoptotic p38 MAPK pathway [75]. Indeed, other sparse pieces of evidence inferring on the potential beneficial effects of PACAP have been previously described in the kidney using* in vitro* models, such as vasodilation and renin secretion [76] and protective effects against hydrogen peroxide-induced oxidative stress [72].* In vivo* models of diabetic nephropathy have also proven the protective role of PACAP in this pathological condition. Its ameliorative effects against high glucose-driven kidney impairment seem to be in part due to inhibition of apoptotic, fibrotic, and oxidative pathways, paralleled by anti-inflammatory properties of the peptide [75, 77]. Although promising, these investigations may be considered as proof-of-concept studies and will require more in-depth studies to really prove the potential therapeutic validity of PACAP against this specific diabetes-associated complication.

## 9. Neuropathy and Future PACAP Application?

A further severe complication in most cases secondary to angiopathy is diabetic neuropathy, which affects the peripheral nervous system. Over half of all patients affected by diabetes develop some form of neuropathy, resulting in sensory loss, pain, and autonomic dysfunction. These manifestations of neuropathy can severely reduce a patient's quality of life. As for the previous complications discussed above, duration of diabetes and glycemic control are important risk factors for neuropathy in both patients with type 1 and type 2 diabetes [78]. Actually, no therapy is approved by regulatory bodies in Europe and the United States for diabetic degenerative neuropathy. The only strategy adopted is the maintenance of normoglycemia, but current treatments for pain management are not effective and/or do not target the causes of diabetes-induced pain. The lack of therapies largely reflects the absence of suitable animal models to study the exact causes of neuropathy [79]. In this scenario, PACAP could be considered a good candidate target considering its numerous beneficial properties. It can circulate in the brain and to some extent cross the blood brain barrier. In particular, the shorter form (PACAP 27) is transported into the brain by transmembrane diffusion, a nonsaturable mechanism, while the uptake of PACAP 38 into the brain occurs through a saturable mechanism. However, to date no significant data has been produced in relationship to this diabetic complication. A plausible reason to explain such an apparent lack of interest on the use of PACAP to treat or prevent diabetic neuropathy could be related to its relatively low bioavailability in the brain. In fact, when administered systemically, native PACAP is rapidly hydrolyzed by the ubiquitous enzyme dipeptidyl-peptidase IV (DPP IV) to form PACAP (3–38) or PACAP (5–38), which are two shorter forms of the peptide with an antagonist activity on PAC1 receptors in most cases [80]. The degradation by DPP IV in the blood circulation also results in the poor metabolic stability with short half-life between 2 and 10 min after PACAP is injected into mice or human [81]. N-terminal truncation of PACAP by removal of the first five amino acids results in a potent PAC1 antagonist that retains the ability to bind PACAP binding sites, does not stimulate adenylate cyclase, and inhibits the ability of PACAP to stimulate adenylate cyclase. With this in mind, the beneficial results of PACAP treatment may be present only when the peptide is administered locally, and this is a great limitation to its clinical use. Current efforts by research laboratories and biotechnologies are now being put to develop a more stable form of the peptide that still retains its ability to cross the blood brain barrier and reach the central or peripheral nervous system. However, at least a research group has succeeded in developing a novel cyclopeptide from the cyclization of PACAP (1–5) that possesses potent activity towards PAC1 and is able to attenuate experimentally-induced diabetes and ganglionic cell death [82, 83], suggesting that advances have been made to overcome the problems associated with poor peptide availability. An alternative objective being pursued is to increase the average half-life of the peptide once systemically injected. The strategy could be by blocking DPP IV enzymatic activity on PACAP through the coadministration of a specific inhibitory agent. Interestingly, metformin, an oral agent commonly prescribed to treat type 2 diabetes involved in the reduction of hepatic glucose production and/or insulin resistance, has shown to also decrease plasma DPP IV activity, limiting the inactivation of exogenously administered peptides [84]. In this regard, Ahrén and Hughes (2005) [85] have elegantly demonstrated that another DDP IV inhibitor, namely, valine-pyrrolidide, when administered through gastric gavage, increases insulin response to PACAP and other substrates of the enzyme, opening new perspectives on the potential clinical validity of the peptide.

## 10. Conclusions

The present review summarizes the findings on the neuroprotective potential of PACAP in major diabetes complications. Based on these data, PACAP emerges as a powerful and promising candidate in the treatment of those pathological conditions associated with long-lasting diabetes. From the present review it arises that diabetic complications are the main consequence of significant changes in vascular structure and function, along with other metabolic alterations. PACAP appears to be able to effectively counteract and/or prevent the majority of these changes, making it a potentially valuable candidate to treat diabetes complication. Unfortunately, a major hurdle that has significantly limited PACAP therapeutic use in T2D-related injuries depends on the rapid hydrolysis of the peptide occurring when injected systemically. Future efforts are therefore necessary to develop a peptide analogue which is more stable, can easily cross human biological barriers (i.e., blood and retinal brain barrier) to bind its receptors in affected organs/systems, and possesses adequate pharmacokinetics. Alternatively, other routes could be to take advantage of new generation viruses for PACAP gene delivery to treat this still unmet medical need, but further studies in this direction are yet to be carried out to exclude any possible associated risk.

## Figures and Tables

**Table 1:** Main complications associated with longstanding diabetes.

Complication	Description
Micro- and macroangiopathy (diabetic endothelial dysfunction)	They are distinctive phenotypes found in both types of diabetes, which are responsible for the high incidence of stroke, heart attack, and organ damage in diabetic patients.

Pancreatic islets dysfunction	It is a progressive dysfunction of pancreatic islet alpha and beta cells, caused either by cell death or dedifferentiation in immature fetal or neonatal-like state with impaired glucose-stimulated insulin secretion which results in inadequate control of hyperglycemia.

Diabetic retinopathy (DR)	It damages the small blood vessels that serve the retina, with loss of visual ability. Further morbidity states associated with DR and diabetes are the increased probability to develop eye diseases such as glaucoma and cataracts.

Diabetic nephropathy	It is a progressive reduction of the filter function of the kidney that, if untreated, can lead to renal failure up to the need of dialysis and/or kidney transplant.

Diabetic neuropathy	It is one of the most frequent complications and according to the World Health Organization is manifested at different levels in 50% of diabetics. It can cause loss of sensitivity, pain of varying intensity, damage to limbs, requiring amputation in more severe cases, and increase in vascular permeability. It may involve heart dysfunction of the eyes and stomach and is a major cause of male impotence.
